# Epidemiological Features of Glycemic Levels and Relative Determinants at Different Altitudes Among Tibetans in China: A Cross-Sectional Population-Based Study

**DOI:** 10.3389/fpubh.2020.00472

**Published:** 2020-09-10

**Authors:** Qiuxing Lin, Jie Liu, Shaopeng Xu, Xianjia Ning, Jun Tu, Qing Yang, Jinghua Wang

**Affiliations:** ^1^Department of Neurology, Tianjin Medical University General Hospital, Tianjin, China; ^2^Laboratory of Epidemiology, Tianjin Neurological Institute, Tianjin, China; ^3^Key Laboratory of Post-Neuroinjury Neuro-repair and Regeneration in Central Nervous System, Ministry of Education and Tianjin City, Tianjin Neurological Institute, Tianjin, China; ^4^Department of Cardiology, Tianjin Medical University General Hospital, Tianjin, China

**Keywords:** altitude, glycemia, epidemiology, risk factors, Tibetans

## Abstract

Risk factors associated with diabetes mellitus have been widely researched worldwide, but the determinants of glycemic levels among Tibetans in China are currently unclear. We thus aimed to determine the relationship between altitude and glycemic levels and to identify factors associated with glycemic levels among Tibetans in China. In 2011, a total of 1,659 Tibetans (aged ≥18 years) from Changdu, China, were enrolled to this cross-sectional research. Potential factors associated with postprandial glucose (PPG), fasting plasma glucose (FPG), and insulin (INS) levels were assessed. FPG and PPG levels increased with age and total cholesterol (TC) level. In addition, FPG levels were higher among patients with rural residence and hypertension, while PPG levels increased with increasing BMI. INS levels increased with residence, lower education, higher BMI, and higher TG levels and decreased with higher altitude and TC levels. Moreover, risk factors for FPG, PPG, and INS differed in those residing at a higher altitude. These findings identify several important risk factors that affect glycemic levels and may be used to develop effective strategies for metabolic disease prevention among populations in high-altitude areas. Furthermore, these findings suggest that it is necessary to formulate a standard for PPG, FPG, and INS in high-altitude areas.

## Introduction

Diabetes mellitus (DM) is one of the most common chronic diseases worldwide, and it continues to increase in prevalence and disease burden (1). Since 1980, the number of adults with diabetes has quadrupled in the world. The burden of DM, both in terms of economic burden and prevalence, has increased more quickly in developing countries than in developed countries (2). In the past two decades, the prevalence of type 2 (T2) DM in China has risen rapidly, and it has become the country with the highest prevalence and number of patients (2, 3).

A few studies have demonstrated the epidemiology of DM and its major risk factors at different altitudes. A recent study reported an odds ratio (OR) of 0.88 for T2 DM among those living 1,500–3,500 m above sea level (4). In addition, some studies reported that residents in high-altitude areas have lower glycemic levels than residents in sea-level areas (5–7).

However, these studies were conducted in populations living at the same altitudes and did not include the influence of ethnic background. Furthermore, some laboratory tests were not included in these studies. Thus, the influence of altitude on glycemic levels among Tibetans, including lamas, residing at different altitudes is unclear.

We thus conducted a population-based survey among Tibetans residing at different altitudes in China. The purpose of this study was to evaluate the relationship among various factors, particularly altitude, on glycemic levels.

## Materials and Methods

### Study Population

This study design has been described previously (8). The survey was conducted from September 2010 to June 2011. The study population was recruited from the Changdu region of the Tibet Autonomous Region of China. There are 11 counties, including 142 townships and 11 central temples, in the Changdu region, with altitudes between 3,200 and 4,500 m. More than 95% of residents are Tibetan.

A total of 1,960 people were selected and invited to participate in the survey. A total of 1,766 people completed the study. The overall response rate was 90.1%. Of those who responded, 1,659 Tibetans were included, after excluding 134 residents without complete demographic data and 167 residents of other ethnicities.

A representative sample of the Tibetan population in China was selected using the multistage (altitude-county-township-village) stratified cluster random sampling method. First, according to different altitude levels (<3,500 m [lower altitude], 3,500–4,000 m [moderate altitude], and >4,000 m [high altitude]), all 11 counties in Changdu were stratified into three groups. Second, one county was selected from each altitude group, which included three counties. Third, four townships from each selected county were selected, for a total of 12 townships. Fourth, three villages or neighborhoods were selected from each selected township. Finally, 36 villages or neighborhoods were selected. Moreover, in each county, we selected one central temple; all qualified lamas were recruited in this study. Finally, all residents aged ≥18 years from the selected 31 villages, 5 neighborhoods, and 3 central temples were recruited in this study.

The ethics committee of Changdu Region People's Hospital, Tibet approved the study, and written informed consent was obtained from all participants during recruitment. All methods were performed in accordance with the relevant guidelines and regulations.

### Information Evaluated in This Study

In 2011, 1,659 Tibetan adults aged ≥18 years were recruited to the present study in Changdu, China. Data collection included survey interviews, clinical measurements, and laboratory tests. Standard questionnaires were conducted by trained researchers to collect information about demographic characteristics, anthropometry, personal and family medical history, educational, and lifestyle risk factors including hypertension, obesity, alcohol consumption, and current smoking. The physical examinations and laboratory tests were completed by professional. The relationship of glycemic level with geographic altitude and demographic characteristics was assessed.

Detailed information was collected with regard to sex, age group (18–34, 35–44, 45–54, 55–64, ≥65 years), education level (0, 1–6, >6 years), socioeconomic status (yearly family income: <800, 800–1,600, ≥1,601 USD/year), cigarette smoking (no, yes), butter tea consumption (no, yes), alcohol use (no, yes), residence (rural/pastoral area, urban area, temple), and altitude (<3,500, ≥3,500 m). In addition to blood pressure measurement, physical examinations also included body height, weight, and circumferences of the waist, hip, and abdomen. Moreover, fasting plasma glucose (FPG), postprandial glucose (PPG), and insulin (INS) levels were tested; Blood lipid-related testing [total cholesterol (TC), triglycerides (TG), low-density lipoprotein-cholesterol (LDL-C), and high-density lipoprotein-cholesterol (HDL-C levels)] was also conducted.

### Definitions

The normal group was defined as having a range of 3.9–6.1 mmol/L for FPG, ≤7.8 mmol/L for 2-h PPG, and 35–145 pmol/L for INS.

Hypertension was defined as an average systolic blood pressure (SBP) of ≥140 mmHg and/or an average diastolic blood pressure (DBP) of ≥90 mmHg, or current use of any medication for treating hypertension within 2 weeks or any combination of the above (9).

Body mass index (BMI) was calculated as the ratio of weight to height squared (kg/m^2^). According to standard criteria in Chinese adults, participants with a BMI ≥24 and <28 kg/m^2^ were classified as overweight, and those with a BMI ≥28 kg/m^2^ were classified as obese (10).

### Data Collection

Data collection was conducted by face-to-face interviews in the community health stations, and physical examinations were performed by investigators who underwent strict training by epidemiology professionals before the start of the study. A few participants completed the survey at home. A preordain standardized questionnaire was administered in this survey.

### Statistical Analysis

Continuous variables are presented as means [standard deviations (SD)], and categorical variables are expressed as percentages [95% confidence intervals (Cis)]. Categorical variables were analyzed using the chi-squared test. The rates of influencing factors, including hypertension, obesity, and smoking, were presented according to five age groups. The risk factors of FPG, PPG, and INS were analyzed by sex, age group, yearly family income, education level, residence, altitude, smoking, butter-tea consumption, and alcohol consumption in the univariate analysis. Determinants of FPG, PPG, and INS were assessed by multivariate linear regression analysis after adjustment for confounding factors that were statistically significant. Statistical significance was defined as a two-tailed *P* < 0.05. Statistical analyses were undertaken using the computer software SPSS (version 15.0 for Windows; SPSS, Chicago, IL, USA).

## Results

### Descriptive Characteristics of Participants

A total of 1,659 people (822 men: 49.5%; 837 women: 49.5%) were selected for this cross-sectional study; 60% of participants were <45 years of age. In addition, 56.4% of participants resided in rural/pastoral areas, and 54.7% lived at an altitude of <3,500 m. The average education level was low, with a relative high overall illiteracy rate of 60%. In addition, 56.9% participants had a yearly family income of <800 USD/year. Moreover, 6.1% of participants smoked, and 8.9% of participants consumed alcohol. The average levels of FPG, PPG, and INS were 5.12, 5.93, and 8.77 mmol/L, respectively (Table 1).

**Table 1:** The demographic characteristics of participants in Tibetans.

**Characteristics**	**Men (*n =* 822)**	**Women (*n =* 837)**	**Total (*n =* 1,659)**
Age, years, means (SD)	41.45 (15.01)	46.51 (15.05)	44.00 (15.24)
**Age group**, ***n*** **(%):**			
18–34 yrs	299 (36.4)	194 (23.2)	493 (29.7)
35–44 yrs	194 (23.6)	193 (23.1)	387 (23.3)
45–54 yrs	161 (19.6)	201 (24.0)	362 (21.8)
55–64 yrs	105 (12.8)	138 (16.5)	243 (14.6)
≥65 yrs	63 (7.7)	111 (13.3)	174 (10.5)
**Education**, ***n*** **(%):**			
0 yrs	394 (47.9)	607 (72.5)	1,001 (60.3)
1–6 yrs	330 (40.1)	102 (12.2)	432 (26.0)
>6 yrs	98 (11.0)	128 (15.3)	226 (13.6)
**Residence**, ***n*** **(%):**			
Rural/pastoral area	353 (43.0)	586 (70.0)	936 (56.4)
Urban	144 (17.5)	251 (30.0)	395 (23.7)
Temple	325 (39.5)	3 (0.4)	328 (19.8)
**Altitude, meter**, ***n*** **(%)**			
<3,500	444 (54.0)	463 (55.3)	907 (54.7)
3,500–4,000	266 (32.4)	334 (39.9)	600 (36.2)
>4,000	112 (13.6)	40 (4.8)	152 (9.1)
**Family income yearly, USD:**			
<800	508 (61.8)	436 (52.1)	944 (56.9)
800–1,600	145 (17.6)	162 (19.4)	307 (18.5)
≥1,601	169 (20.6)	239 (28.5)	408 (24.6)
Current smoking, *n* (%)	73 (11.2)	6 (0.9)	79 (6.1)
Alcohol consumption, *n* (%)	48 (7.4)	67 (10.5)	115 (8.9)
FPG, mmol/L, means (SD)	5.12 (1.77)	5.13 (1.52)	5.12 (1.65)
PPG, mmol/L, means (SD)	5.89 (3.31)	5.97 (2.80)	5.93 (3.07)
INS, mmol/L, means (SD)	8.79 (6.31)	8.75 (5.86)	8.77 (6.10)

### Factors Associated With Blood Glucose Levels in the Univariate Analysis

**Figure 1:**
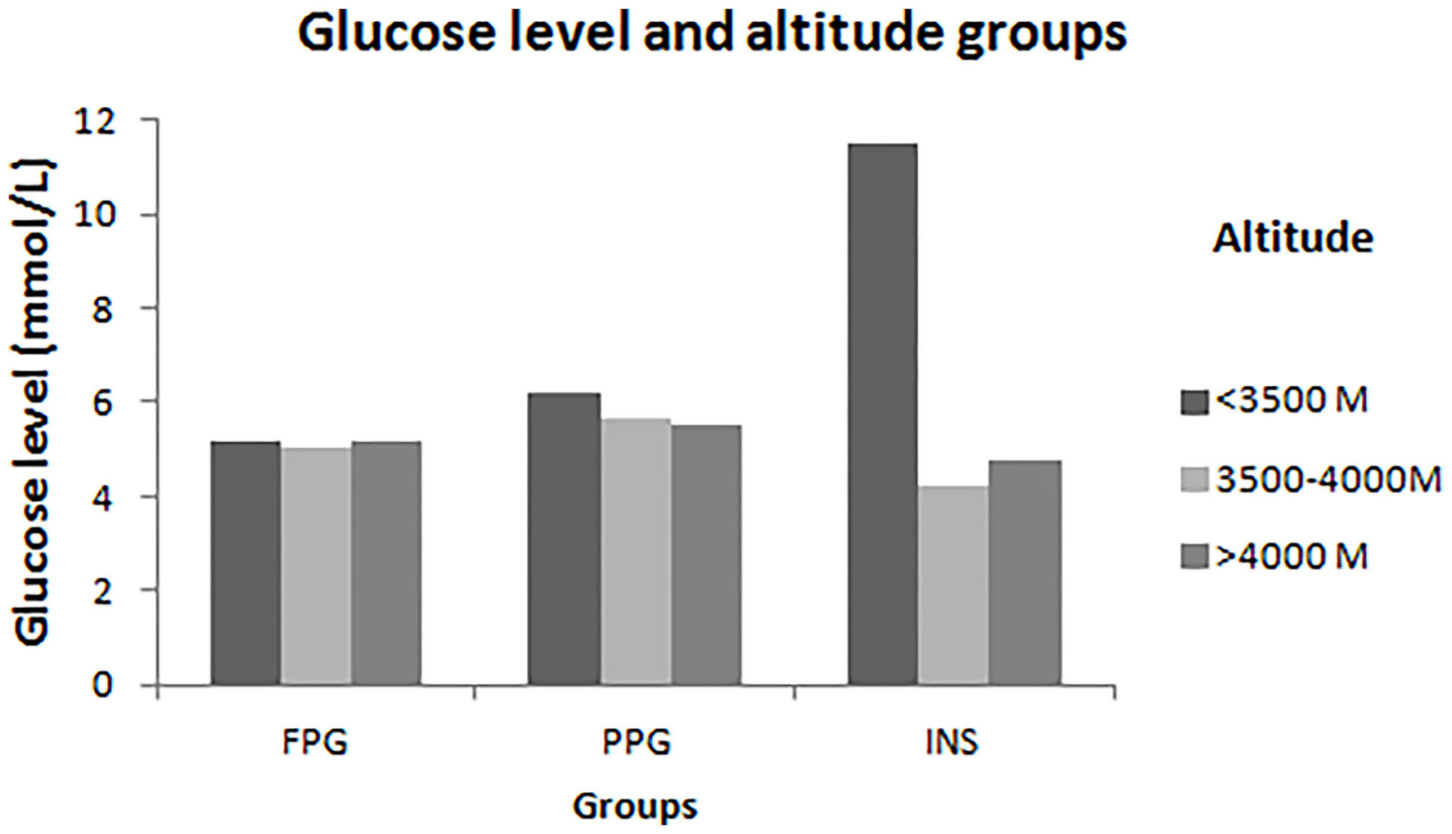
Levels of FPG, PPG and INS in different altitude. This figure showed that there are significantly different risk factors to FPG, PPG and INS in different altitude levels among Tibetans aged ≥18 years in China. In the lower-altitude level (<3,500 m), age, hypertension, residence and TG were risk factors of FPG; age and TG were risk factors of PPG; and residence was the risk factor of INS. In the middle- altitude level (3,500–4,000 m), gender, TC, and LDL were associated with PPG; age, education, drinking, TG, and LDL were associated with PPG. In high-altitude level (>4,000 m), TG was the only one risk factor of FPG and PPG.

FPG, PPG, and INS levels were associated with residence, hypertension, and BMI; furthermore, FPG and PPG levels increased with age group. PPG and INS levels were associated with altitude (Figure 1). INS levels were 2.41-fold higher among low-altitude residents than among high-altitude residents. In addition, alcohol consumption significantly increased PPG levels by 1.12-fold compared to non-alcohol consumption. INS levels were also related to education level, smoking, and consuming butter tea (Table 2). Moreover, FPG and PPG levels were increased with increasing TC, TG, and LDL; INS was inversely proportional to LDL (Table 3).

**Table 2:** Levels of blood glucose in the different groups by demography.

**Characteristics**	**FPG**	**PPG**	**INS**
Gender:	0.897	0.645	0.934
Men	5.12 (1.77)	5.89 (3.31)	8.79 (6.31)
Women	5.13 (1.52)	5.97 (2.80)	8.75 (5.86)
Age group:	<0.001	<0.001	0.141
18–34 yrs	4.73 (0.69)	5.02 (1.41)	7.90 (5.07)
35–44 yrs	5.00 (1.11)	5.66 (2.27)	9.41 (7.15)
45–54 yrs	5.46 (2.65)	6.44 (4.17)	8.51 (4.93)
55–64 yrs	5.58 (2.03)	7.22 (4.17)	10.04 (8.01)
65–74 yrs	5.37 (1.26)	6.69 (3.03)	8.64 (4.80)
Education:	0.378	0.746	<0.001
0 yrs	5.10 (1.44)	5.91 (2.71)	7.53 (6.40)
1–6 yrs	5.10 (1.97)	5.90 (3.56)	8.81 (5.85)
>6 yrs	5.28 (1.83)	6.10 (3.48)	11.10 (5.12)
Residence:	<0.001	<0.001	<0.001
Rural/pastoral area	5.10 (1.08)	5.88 (2.49)	3.75 (2.60)
Urban	5.45 (2.36)	6.52 (4.00)	11.04 (5.36)
Temple	4.79 (1.78)	5.36 (3.02)	8.77 (7.22)
Altitude:	0.708	0.007	<0.001
<3,500 m	5.15 (1.94)	6.18 (3.44)	11.53 (5.96)
3,500–4,000 m	5.07 (0.84)	5.66 (1.97)	4.23 (2.92)
>4,000 m	5.16 (2.14)	5.53 (3.86)	4.78 (3.00)
Family income yearly:	0.107	0.008	0.064
<800 USD	5.08 (1.68)	5.72 (3.06)	9.58 (6.71)
800–1,600 USD	5.04 (1.70)	5.98 (2.38)	8.48 (5.54)
>1,600 USD	5.29 (1.56)	6.37 (3.47)	8.05 (5.65)
Hypertension:	<0.001	<0.001	<0.001
Yes	5.35 (1.98)	3.39 (3.57)	9.56 (6.18)
No	4.73 (0.70)	5.14 (1.61)	7.37 (4.83)
BMI groups:	<0.001	<0.001	<0.001
Normal	4.96 (1.25)	5.48 (2.11)	6.88 (4.48)
Over weight	5.28 (1.42)	6.40 (3.43)	10.17 (5.42)
Obesity	5.57 (2.83)	7.12 (6.86)	12.42 (8.01)
Current smoking	0.505	0.542	0.012
Yes	5.00 (1.00)	5.73 (2.55)	11.14 (5.90)
No	5.13 (1.69)	5.95 (3.10)	8.55 (6.07)
Alcohol consumption	0.172	0.001	0.064
Yes	5.32 (1.26)	6.81 (3.02)	7.35 (5.52)
No	5.10 (1.69)	5.84 (3.06)	8.97 (6.15)
Butter tea consumption	0.576	0.783	0.028
Yes	5.13 (1.66)	5.93 (3.09)	8.61 (6.16)
No	5.00 (1.24)	6.05 (2.55)	11.18 (4.49)

**Table 3:** The association of different glucose level with measurements of lipids in the univariate analysis.

**Risk factors**	**FPG**		**PPG**		**INS**	
	**β (95% CI)**	***P***	**β (95% CI)**	***P***	**β (95% CI)**	***P***
TC	0.321 (0.240, 0.403)	<0.001	0.647 (0.494, 0.801)	<0.001	0.717 (0.190, 1.244)	0.008
TG	0.377 (0.302, 0.451)	<0.001	0.642 (0.502, 0.781)	<0.001	1.247 (0.766, 1.728)	<0.001
HDL-C	0.055 (−0.149, 0.260)	0.596	−0.013 (−0.398, 0.373)	0.949	2.188 (0.867, 3.509)	0.001
LDL-C	0.251 (0.137, 0.365)	<0.001	0.597 (0.383, 0.811)	<0.001	−0.652 (−1.406, 0.102)	0.090

### Risk Factors for High FPG, PPG, and INS

Table 4 shows that age group, residence, hypertension, and TG level were significantly associated with FPG among Tibetans aged ≥18 years in the multivariate linear regression analysis, with standard partial regression coefficient (β) [95% confidence intervals (CIs)] of 0.09 (0.02, 0.17; *P* = 0.020) for age group, −0.27 (−0.42, −0.12; *P* < 0.001) for residence, 0.27 (0.07, 0.48; *P* = 0.010) for hypertension, and 0.29 (0.20, 0.38; *P* < 0.001) for higher TG levels after adjustment for confounding factors. Age group, BMI group, and TG were also risk factors for PPG, with β values (95% CIs) of 0.33 (0.19, 0.47; *P* < 0.001) for age group, 0.45 (0.22, 0.68; *P* < 0.001) for BMI group, and 0.46 (0.29, 0.62; *P* < 0.001) for higher TG levels. Furthermore, residence, altitude group, education, BMI group, and TC and TG levels were risk factors for high INS levels. The corresponding β values (95% CIs) of INS were 1.23 (0.41, 2.06; *P* = 0.004) for residence group, −4.39 (−5.39, −3.39; *P* < 0.001) for altitude group, 0.68 (0.07, 1.29; *P* =0.030) for education group, and 2.14 (1.51, 2.77; *P* < 0.001) for BMI group. Moreover, the corresponding β values (95% CIs) of INS were −0.64 (−1.20, 0.78; *P* = 0.026) and 0.57 (0.07, 1.06; *P* = 0.024) for TC and TG levels, respectively.

**Table 4:** Determinants of FPG, PPG, and INS among Tibetans aged ≥18 years using multivariate regression analysis.

**Category**	**FPG**		**PPG**		**INS**	
	**β (95% CI)**	***P***	**β (95% CI)**	***P***	**β (95% CI)**	***P***
Age group	0.09 (0.02, 0.17)	0.020	0.33 (0.19, 0.47)	<0.001	—	—
Altitude group	—	—	−0.01 (−0.29, 0.27)	0.948	−4.39 (−5.39, −3.39)	<0.001
Residence	−0.27 (−0.42, −0.12)	<0.001	−0.24 (−0.54, 0.07)	0.135	1.23 (0.41, 2.06)	0.004
Family income yearly	—	—	0.07 (−0.14, 0.29)	0.518	—	—
Education	—	—	—	—	0.68 (0.07, 1.29)	0.030
Hypertension	0.27 (0.07, 0.48)	0.010	0.35 (−0.03, 0.74)	0.073	−0.22 (−1.1, 1.0)	0.692
BMI group	0.118 (−0.01, 0.24)	0.060	0.45 (0.22, 0.68)	<0.001	2.14 (1.51, 2.77)	<0.001
Current smoking	—	—	—	—	1.17 (−0.63, 2.87)	0.211
Alcohol consumption	—	—	0.38 (−0.20, 0.96)	0.202	—	—
TC	0.15 (−0.03, 0.33)	0.108	0.03 (−0.34, 0.34)	0.985	−0.64 (−1.20, 0.78)	0.026
TG	0.29 (0.20, 0.38)	<0.001	0.46 (0.29, 0.62)	<0.001	0.57 (0.07, 1.06)	0.024
HDL-C	—	—	—	—	0.67 (−0.78, 2.11)	0.366
LDL-C	−0.06 (−0.29, 0.17)	0.590	0.23 (−0.20, 0.65)	0.295	—	—

Based on the interaction results, we did a multivariate analysis according to different altitude groups. In the low altitude group, FPG levels increased in those individuals with high TG level; but decreased by 0.309 mmol/L in individuals lived in rural/pastoral area than those lived in urban and decreased by 0.309 mmol/L in individuals lived in temple than those lived in rural/pastoral area (β, −0.309; 95% CI, −0.514 to −0.105; *P* < 0.001). Moreover, elevated TG level associated with increased FPG (β, 0.251; 95% CI, 0.137–0.365; *P* < 0.001) and PPG (β, 0.495; 95% CI, 0.284–0.705; *P* < 0.001). In the 3,500–4,000 m group, each 1 mmol/L increasing with TG levels resulted in 0.295 mmol/L increase of PPG (β, 0.295; 95% CI, 0.054–0.537; *P* = 0.017). However, in the >4,000-m group, each 1 mmol/L increase of TG level, FPG levels increased by 0.907 mmol/L (β, 0.907; 95% CI, 0.111–1.703; *P* = 0.026), while PPG levels increased by 1.703 mmol/L (β, 1.703; 95% CI, 0.283–3.123; *P* = 0.019) (Table 5).

**Table 5:** Determinants of FPG, PPG and INS at different altitude group.

**Risk factors**	**FPG**		**PPG**		**INS**	
	**β (95% CI)**	***P***	**β (95% CI)**	***P***	**β (95% CI)**	***P***
**<3,500 m:**						
Gender (categorized variable)	—	—	—	—	0.063 (−1.535, 1.661)	0.983
Age (categorized variable)	0.186 (0.690, 0.302)	0.002	0.436 (0.223, 0.649)	<0.001	—	—
Education (categorized variable)	—	—	—	—	—	—
**Hypertension (categorized variable)**						
	0.480 (0.158, 0.802)	0.004	0.538 (−0.056, 1.132)	0.076	—	—
**Residence (categorized variable)**						
	−0.309 (−0.514, −0.105)	0.003	−0.153 (−0.511, 0.205)	0.402	1.897 (0.723, 3.070)	0.002
TG (continuous variable)	0.251 (0.137, 0.365)	<0.001	0.495 (0.284, 0.705)	<0.001	0.688 (0.152, 1.244)	0.012
TC (continuous variable)	–0.075 (−0.224, 0.075)	0.327	0.108 (−0.367, 0.583)	0.655	—	—
**3500–4,000 m:**						
Gender (categorized variable)	0.265 (0.117, 0.413)	<0.001	—	—	—	—
Age (categorized variable)	—	—	0.186 (0.256, 1.384)	0.005	—	—
Education (categorized variable)	—	—	−0.432 (−0.801, −0.062)	0.022	—	—
**Hypertension (categorized variable)**						
	0.105 (−0.044, 0.253)	0.167	0.323 (−0.092, 0.738)	0.127	—	—
**Residence (categorized variable)**						
	—	—	−0.336 (−0.819, 0.147)	0.173	—	—
TG (continuous variable)	0.012 (−0.104, 0.128)	0.835	0.295 (0.054, 0.537)	0.017	—	—
TC (continuous variable)	0.505 (0.240, 0.771)	<0.001	−0.223 (−0.637, 0.190)	0.289	—	—
**>4000 m:**						
Gender (categorized variable)	—	—	—	—	−0.754 (−3.835, 2.327)	0.625
Age (categorized variable)	—	—	—	—	—	—
Education (categorized variable)	—	—	—	—	1.322 (−1.218, 3.861)	0.300
**Hypertension (categorized variable)**						
	0.369 (−0.452, 1.191)	0.375	0.789 (-0.677, 2.255)	0.289	—	—
Residence (categorized variable)						
	—	—	—	—	1.658 (-2.256, 5.571)	0.398
TG (continuous variable)	0.907 (0.111, 1.703)	0.026	1.703 (0.283, 3.123)	0.019	—	—
TC (continuous variable)	−0.184 (−1.252, 0.884)	0.734	0.094 (−1.814, 2.003)	0.922	—	—

## Discussion

This is the first study to report factors associated with glycemic levels among Tibetans aged ≥18 years in China. The results of the present study reveal that altitude was an important factor related to glycemic levels. Furthermore, there were significantly different risk factors for FPG, PPG, and INS levels at different altitude levels. At lower altitude (<3,500 m), age, hypertension, residence, and TG levels were risk factors for FPG levels; age and TG levels were risk factors for PPG levels; and residence was a risk factor for INS levels. At moderate altitude (3,500–4,000 m), sex, TC, and LDL were risk factors for PPG levels; and age, education, drinking, and TG and LDL levels were risk factors for PPG levels. At high altitude (>4,000 m), TG levels were the only risk factor for FPG and PPG levels; in contrast, after adjusting for other conventional risk factors, there were no risk factors for INS levels at moderate or high altitude.

Risk factors for FGP, PPG, and INS levels at lower altitude (<3,500 m) were similar to those of previous studies reporting risk factors at sea level. However, the current study found fewer risk factors in the study population of Tibetans in China. In previous studies, age, obesity, hypertension, TC levels, TG levels, living in low-income areas, alcohol consumption were confirmed as risk factors for DM at sea level (11–14). With regard to age, FPG levels increase with age, with a 0.07-mmol/L increase in as a risk factor FPG levels each decade of life (11). Poor recognition of disease prevention, unawareness of the diabetes and relatively limited medical resources in rural areas probably was the reason of regional difference. In the same time, more than 50% of DM patients had hypertension (12, 13). With regard to residential area as a risk factor, the prevalence of DM in rural areas is higher than that in urban areas in north China (15). These regional differences are likely explained by poor recognition of disease prevention, unawareness of DM, and relatively limited medical resources in rural areas.

There is compelling evidence that populations living at an altitude of 3,000–4,500 m have lower FPG levels than those living below 500 m (16–20). For example, residents living at 3,200 m had lower glycemic levels compared with those living at sea level (5). In addition, a study reported that median FPG level was 81.6 mg/dL for men living above 3,000 m and was significantly higher than that in non-pregnant adult women, who had a median FPG level of 71.7 mg/dL (19); thus, there are also sex differences in FPG levels at this altitude. Moreover, sex was closely related to the postprandial glucose response in patients with T2 DM in low-altitude (14). Thus, the relationship between sex and FPG in the present study is consistent with findings of previous studies.

It is noteworthy that risk factors for high FPG levels in residents living at moderate altitude (3,500–4,000 m) included sex and TC levels. Moreover, age, education, and TC were negatively related with PPG levels in residents living at moderate altitude. Furthermore, alcohol consumption was an independent risk factor for PPG, after adjusting for other conventional risk factors. This is consistent with previous research, which showed that excessive drinking was related to elevated FPG levels in adults aged 16–43 years (21). The relationship of alcohol consumption and the incidence of DM has been widely accepted, and alcohol consumption exhibits a U-shaped relationship with the risk of T2 DM (22, 23). There were no risk factors.

At high altitude (>4,000 m), TG levels were the only independent risk factor for FPG and PPG levels, after adjustment for other conventional risk factors.

According to above findings, it is evident that there are fewer risk factors for hyperglycemia in residents at increasing altitudes. However, as the study population were residents, these findings may not apply to the short-term effects of altitude on blood glucose levels. Fasting glucose levels of healthy people living at sea level exposed to very high altitude has been reported to increase, decrease, or remain unchanged (24–26). Moreover, a recent study reported that people have better glycemic control at higher altitudes (27). In contrast, another study reported no significant differences in fasting glycemia between low and high altitudes (28). Nonetheless, the present study in Tibetans reveals important insights regarding the effects of altitude on risk factors for high glycemic levels in residents.

There were several limitations in this study. First, the population was only selected from the Changdu region; however, the four-stage randomly stratified cluster sampling method was used to make the study population representative. Second, a lower altitude (closer to sea level) population was not included in this comparison, as all participants lived above 3,200 m; however, the subgroup analyses were stratified by altitude (<3,500 and ≥3,500 m). Third, dietary intake and physical activity were not considered in this study; however, some lifestyle factors, including smoking, alcohol consumption, and butter tea consumption were evaluated. Finally, we did not use hemoglobin A1c level as a biomarker of glycemia, and this may result in less stable findings regarding the glycemic state. Future studies should include a more stable indicator of glycemia.

This is the first study to report factors influencing glycemic level among Tibetans aged ≥18 years in China. Risk factors differed by altitude levels among Tibetans in China. FPG levels were associated with age, hypertension, rural residence, and TG levels in residents at lower altitude (<3,500 m); with sex, TC levels in residents at moderate altitude (3,500–4,000 m); and only with TG levels in residents at high altitude (>4,000 m). Similarly, PPG levels were associated with age and TG levels at lower altitude (<3,500 m); with age, education, and TG levels at moderate altitude (3,500–4,000 m); but only TG level at high altitude (>4,000 m). Moreover, INS levels were associated with residence and TG levels only at lower altitude (<3,500 m). These findings suggest that management of blood glucose levels should be approached differently according to different altitude levels.

## Data Availability Statement

All datasets generated for this study are included in the article/supplementary material.

## Ethics Statement

The studies involving human participants were reviewed and approved by the ethics committee of Changdu Region People's Hospital. The patients/participants provided their written informed consent to participate in this study.

## Author Contributions

JW, QY, and SX were involved in conception and design, data collection, data interpretation, and critical review for this article. JW was involved in data analysis for this article. QL and JL were involved in manuscript drafting. QL, JL, SX, XN, JT, QY, and JW were involved in data collection, case diagnosis, and confirmation for this article. All authors reviewed the manuscript.

## Conflict of Interest

The authors declare that the research was conducted in the absence of any commercial or financial relationships that could be construed as a potential conflict of interest.
